# UVB Radiation and Selected Tryptophan-Derived AhR Ligands—Potential Biological Interactions in Melanoma Cells

**DOI:** 10.3390/ijms22147500

**Published:** 2021-07-13

**Authors:** Katarzyna Walczak, Paulina Kazimierczak, Karolina Szalast, Tomasz Plech

**Affiliations:** 1 Department of Pharmacology, Medical University of Lublin, Chodźki 4a, 20093 Lublin, Poland; karolina.szalast@umlub.pl (K.S.); tomasz.plech@umlub.pl (T.P.); 2 Department of Biochemistry and Biotechnology, Medical University of Lublin, Chodźki 1, 20093 Lublin, Poland; paulina.kazimierczak@umlub.pl

**Keywords:** aryl hydrocarbon receptor, UVB, kynurenine, kynurenic acid, tryptophan, melanoma, tumor cell transendothelial cell migration assay, necrosis, proliferation

## Abstract

Excessive UV exposure is considered the major environmental factor in melanoma progression. Human skin is constantly exposed to selected tryptophan-derived aryl hydrocarbon receptor (AhR) ligands, including kynurenine (KYN) and kynurenic acid (KYNA), as they are endogenously produced and present in various tissues and body fluids. Importantly, recent studies confirmed the biological activity of KYN and KYNA toward melanoma cells in vitro. Thus, in this study, the potential biological interactions between UVB and tryptophan metabolites KYN and KYNA were studied in melanoma A375, SK-MEL-3, and RPMI-7951 cells. It was shown that UVB enhanced the antiproliferative activity of KYN and KYNA in melanoma cells. Importantly, selected tryptophan-derived AhR ligands did not affect the invasiveness of A375 and RPMI-7951 cells; however, the stimulatory effect was observed in SK-MEL-3 cells exposed to UVB. Thus, the effect of tryptophan metabolites on metabolic activity, cell cycle regulation, and cell death in SK-MEL-3 cells exposed to UVB was assessed. In conclusion, taking into account that both UVB radiation and tryptophan-derived AhR ligands may have a crucial effect on skin cancer formation and progression, these results may have a significant impact, revealing the potential biological interactions in melanoma cells in vitro.

## 1. Introduction

Epidemiological studies revealed that excessive UV exposure is the major environmental factor involved in the development of melanoma [1]. UVB is considered to be more carcinogenic than UVA due to its ability to promote the formation of cyclobutane pyrimidine dimers (CPD) and 6-pyrimidine 4-pyrimidone photoproducts [2]. However, UV radiation also has indirect effects mediated by the reactive oxygen species (ROS) level. Increased ROS production may be involved in several processes during melanomagenesis, including malignant transformation, DNA damage-induced mutations, alteration of the activity of the pro-survival pathways, inflammation, and immunosuppression [3].

Tryptophan, an essential amino acid, is the strongest natural near-UV-absorbing chromophore. Moreover, tryptophan may be transformed into enzymatic and non-enzymatic reactions in many biologically active metabolites, which may directly and indirectly affect skin physiology (reviewed in [4]). Importantly, tryptophan metabolites, such as kynurenine (KYN) and kynurenic acid (KYNA), represent a group of ligands for aryl hydrocarbon receptor (AhR), which is involved in various physiological and pathological processes in the skin [5,6,7,8]. KYN is a key metabolite of the main route of tryptophan catabolism with evidenced immunosuppressive activity [8]. Moreover, previous studies revealed that KYN may promote cancer cell survival and motility by interaction with AhR [7]. However, the direct activity of KYN on cancer cell proliferation has not been fully studied. On the contrary, another tryptophan-derived AhR ligand, KYNA, enzymatically formed from KYN, has antiproliferative and antimigratory properties against various types of cancer cells, including glioma, colon, and renal cancer cells [9,10,11,12,13,14].

Human skin is constantly exposed to tryptophan-derived AhR ligands KYN and KYNA. They are endogenously produced and present in various tissues and body fluids (reviewed in [4,15,16]). Moreover, these tryptophan metabolites are present in various herbs, bee products, and vegetables, which not only are components of the human daily diet but may also be used as natural extracts in skin care and body treatments [17,18,19,20,21,22]. Additionally, previous studies revealed that KYNA is absorbed from the gastrointestinal tract and transported with the blood to peripheral tissues [17,21,22].

Interestingly, recent research has revealed that selected tryptophan-derived AhR ligands, including KYN and KYNA, possess biological activity toward melanoma A375 and RPMI-7951 cells [11]. The tryptophan metabolites affected DNA synthesis, cell cycle regulators, and cell death of melanoma cells in vitro. However, despite belonging to the same group of tryptophan-derived AhR ligands, KYN and KYNA had different activities and molecular mechanisms toward A375 and RPMI-7951 cells [11].

Considering that both UVB radiation and tryptophan-derived AhR ligands may be involved in the induction and progression of skin cancer, the main aim of this study was to assess the potential biological interactions between UVB and selected tryptophan-derived AhR ligands, KYN and KYNA, in human melanoma cells in vitro. In this study, we focused on the effect of UVB, KYN and KYNA on the proliferation and migration of human melanoma A375, SK-MEL-3 and RPMI-7951 cells.

## 2. Results

**Figure 1:**
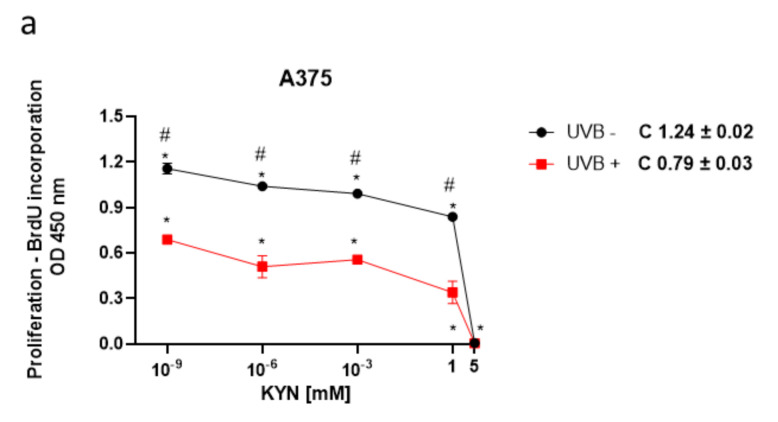

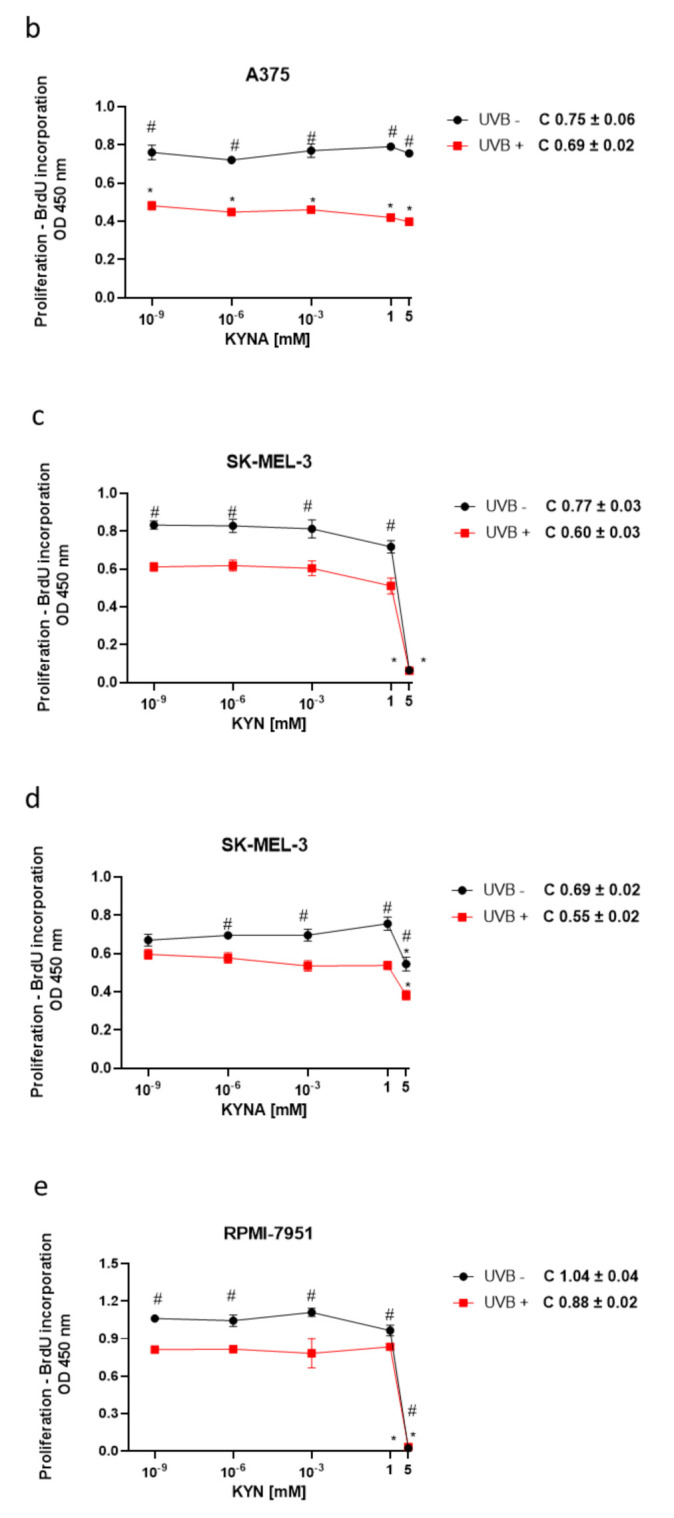

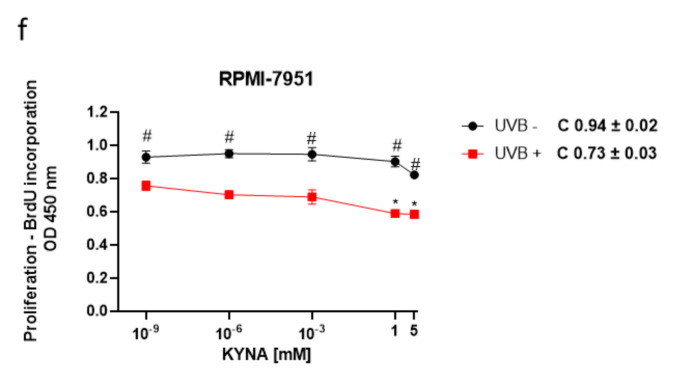
The effect of UVB on the proliferation of melanoma A375 (**a**,**b**), SK-MEL-3 (**c**,**d**), and RPMI-7951 (**e**,**f**) cells exposed to KYN and KYNA. Melanoma A375, SK-MEL-3, and RPMI-7951 cells were exposed to UVB (UVB+) and then to culture medium (control, C) or serial dilutions of KYN and KYNA (10^−9^, 10^−6^, 10^−3^, 1, and 5 mM) for 24 h. Similarly, antiproliferative activity of KYN and KYNA was studied in melanoma cells under standard conditions (UVB−). The effect of UVB and tested substances on proliferation (DNA synthesis) of melanoma cells was assessed by the BrdU Assay. Data represent the mean value ± SEM of eight independent experiments. Values significant (*) in comparison with the appropriate control (UVB− or UVB+) with *p* < 0.05 (one-way ANOVA with Tukey’s post hoc test). Values significant (#) UVB− vs. UVB+ with *p* < 0.05 (unpaired *t*-test). The effect of KYN and KYNA on BrdU incorporation in A375 and RPMI-7951 cells under standard conditions has been previously published in [11].

The BrdU assay was used to assess the biological activity of KYN and KYNA on melanoma cell proliferation upon UVB treatment. The effect of KYN and KYNA on DNA synthesis was studied in human melanoma A375, SK-MEL-3, and RPMI-7951 cells, representing different stages of melanomagenesis, under standard conditions and after exposure to UVB (Figure 1). KYN inhibited DNA synthesis in all melanoma cell lines under standard conditions. The inhibitory effect in A375 cells was already observed at a concentration of 10^−9^ mM (Figure 1a). SK-MEL-3 and RPMI-7951 cell lines, derived from melanoma metastasis, were more resistant to antiproliferative activity of KYN; however, KYN at a concentration of 5 mM significantly inhibited DNA synthesis in these cell lines (Figure 1c,e). KYNA at a concentration of 5 mM moderately reduced DNA synthesis (20.7%) only in SK-MEL-3 cells under standard conditions (Figure 1d). Importantly, UVB itself inhibited DNA synthesis in melanoma cells and acted synergistically with selected tryptophan metabolites. Moreover, UVB enhanced the inhibitory activity of KYNA in A375 and RPMI-7951 cells (Figure 1b,f).

**Figure 2:**
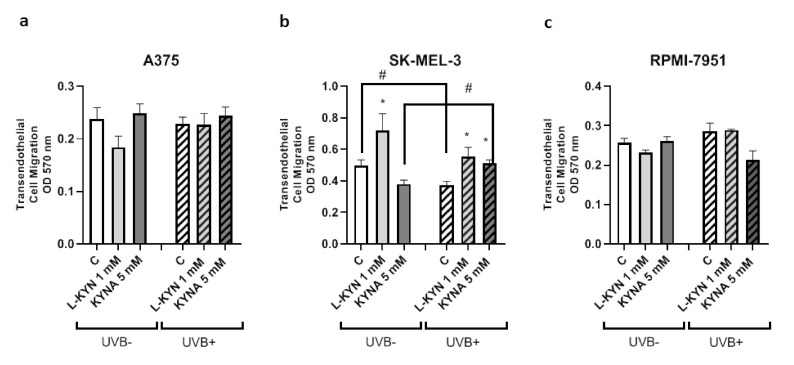
The effect of UVB on the invasiveness and motility of melanoma A375 (**a**), SK-MEL-3 (**b**) and RPMI-7951 (**c**) cells exposed to KYN and KYNA. Melanoma A375, SK-MEL-3, and RPMI-7951 cells were exposed to UVB and then to culture medium (control, C) or dilutions of KYN (1 mM) or KYNA (5 mM) for 24 h. Similarly, the biological activity of KYN and KYNA was studied in melanoma cells under standard conditions (UVB−). The ability of melanoma cells to invade the endothelium (HUVEC) was assessed by the Tumor Cell Transendothelial Migration Assay. Data represent the mean value ± SEM of three independent experiments. Values significant (*) in comparison with appropriate control (UVB− or UVB+) with *p* < 0.05 (unpaired *t*-test). Values significant (#) UVB− vs. UVB+ with *p* < 0.05 (unpaired *t*-test).

To determine the effect of UVB on the invasiveness and motility of melanoma cells exposed to KYN and KYNA, the Tumor Cell Transendothelial Migration Assay was utilized. KYN and KYNA did not affect the invasiveness of A375 and RPMI-7951 cells under standard conditions and after exposure to UVB (Figure 2a,c). Interestingly, KYN at a concentration of 1 mM stimulated the invasiveness and motility of melanoma SK-MEL-3 cells through endothelial monolayer both under standard conditions and after exposure to UVB (Figure 2b). On the other hand, KYNA at a concentration of 5 mM enhanced the migration of SK-MEL-3 cells only after exposure to UVB (Figure 2b). Additionally, UVB itself reduced the invasiveness of melanoma SK-MEL-3 cells (Figure 2b).

**Figure 3:**
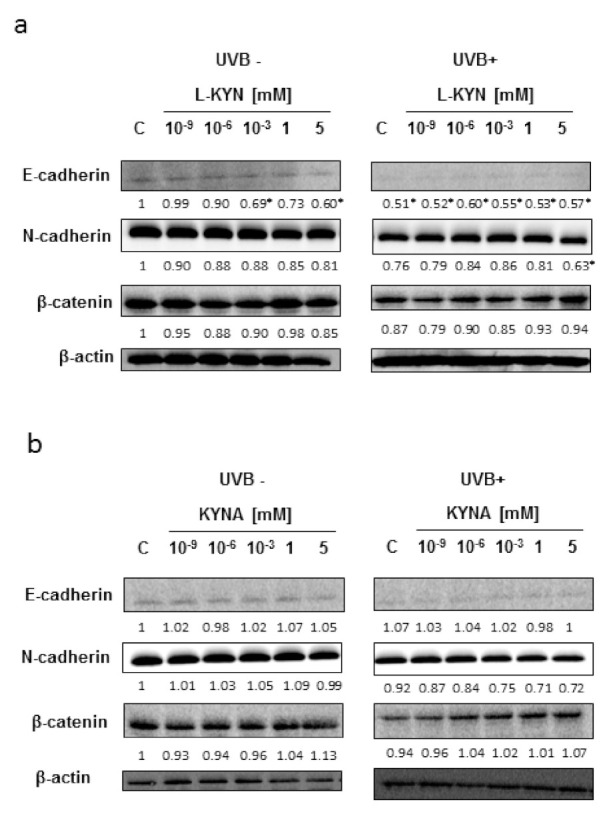
The effect of UVB on E-cadherin, N-cadherin, and β-catenin levels in melanoma SK-MEL-3 cells exposed to KYN (**a**) and KYNA (**b**). Melanoma SK-MEL-3 cells were exposed to UVB (UVB+) and then to culture medium (control, C) or serial dilutions of KYN and KYNA (10^−9^, 10^−6^, 10^−3^, 1, and 5 mM). Similarly, the biological activity of KYN and KYNA was studied in melanoma cells under standard conditions (UVB−). The effect of UVB and tested substances on the protein level of selected proteins involved in adhesion and migration (E-cadherin, N-cadherin, and β-catenin) was determined by means of Western blot. Western blots were selected as the most representative of the series of repetitions. UVB− and UVB+ bands represent the same blot. The results of densitometric analysis, normalized relative to β-actin, are shown as relative value of the control (“*”, the fold changes in protein expression ≥30% were considered significant; qualitative analysis).

To reveal the potential molecular mechanism of this phenomenon, we studied the effect of UVB, KYN, and KYNA on the expression of proteins involved in adhesion (E-cadherin, N-cadherin, and β-catenin) in SK-MEL-3 cells. The protein level of E-cadherin in melanoma SK-MEL-3 was low under standard conditions and after exposure to UVB (Figure 3a,b). KYN and KYNA did not significantly affect the protein level of tested cadherins and β-catenin in SK-MEL-3 cells under standard conditions and in melanoma cells exposed to UVB (Figure 3a,b).

Since our investigation into the proteins involved in adhesion and metastasis did not pinpoint the specific mechanism that could explain the observed changes in the invasiveness and motility of SK-MEL-3 cells, we studied whether UVB, KYN, and KYNA initiate other unexpected changes in metabolic activity, cell cycle regulation, and cell death in SK-MEL-3 cells.

**Figure 4:**
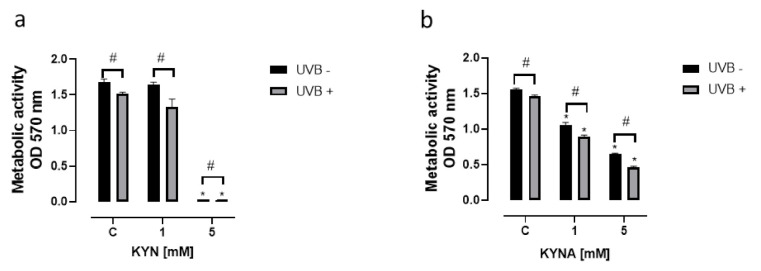
The effect of UVB on the metabolic activity of melanoma SK-MEL-3 cells exposed to KYN (**a**) and KYNA (**b**). Melanoma SK-MEL-3 cells were exposed to UVB (UVB+) and then to culture medium (control, C) or serial dilutions of KYN and KYNA (1 and 5 mM) for 96 h. Similarly, the biological activity of KYN and KYNA was studied in melanoma cells under standard conditions (UVB−). The effect of UVB and tested substances on the metabolic activity of melanoma SK-MEL-3 cells was assessed by the MTT Assay. Data represent the mean value ± SEM of eight independent experiments. Values significant (*) in comparison with appropriate control (UVB− or UVB+) with *p* < 0.05 (one-way ANOVA with Tukey’s post hoc test). Values significant (#) UVB− vs. UVB+ with *p* < 0.05 (unpaired *t*-test).

KYN and KYNA inhibited not only DNA synthesis but also the metabolic activity of melanoma SK-MEL-3 cells (Figure 4). The effect of KYN and KYNA on mitochondrial activity was determined using the MTT Assay. KYN at a concentration of 5 mM reduced metabolic activity by over 98% (Figure 4a), whereas KYNA at a concentration of 1 and 5 mM inhibited the metabolic activity of SK-MEL-3 cells by 32% and 58%, respectively (Figure 4b). The effect was stronger in melanoma cells exposed previously to UVB.

**Figure 5:**
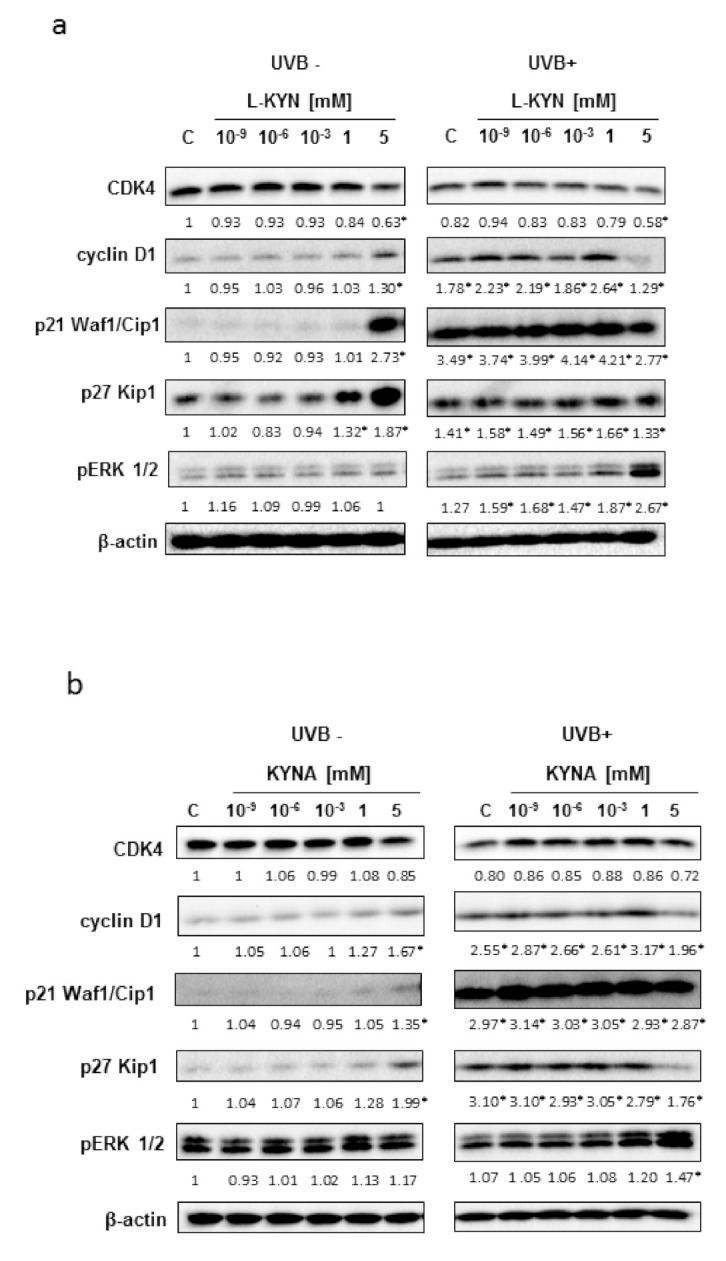
The effect of UVB on selected cell cycle regulators and activation of the ERK signaling pathway in melanoma SK-MEL-3 exposed to KYN (**a**) and KYNA (**b**). Melanoma SK-MEL-3 cells were exposed to UVB (UVB+) and then to culture medium (control, C) or serial dilutions of KYN and KYNA (10^−9^, 10^−6^, 10^−3^, 1, and 5 mM) for 24 h. Similarly, the biological activity of KYN and KYNA was studied in melanoma cells under standard conditions (UVB−). The effect of UVB and tested substances on the protein level of selected cell cycle regulators (cyclin D1, CDK4, p21 Waf1/Cip1, and p27 Kip1) and activation of ERK1/2 kinase was determined by means of Western blot. Western blots were selected as the most representative of the series of repetitions. UVB− and UVB+ bands represent the same blot. The results of densitometric analysis, normalized relative to β-actin, are shown as relative value of the control (“*”, the fold changes in protein expression ≥ 30% were considered significant; qualitative analysis).

To reveal the potential molecular mechanism of the tested tryptophan metabolites in melanoma cells, we studied the effect of KYN and KYNA on the selected cell cycle regulators in SK-MEL-3 cells before and after UVB exposure. KYN decreased the protein level of CDK4 and increased cyclin D1 in melanoma SK-MEL-3 cells (Figure 5a). Similarly, KYNA upregulated the protein level of cyclin D1, but no significant changes in CDK4 protein level were observed (Figure 5b). Both the tested tryptophan metabolites induced the protein expression of cyclin-dependent kinase inhibitors p21 Waf1/Cip1 and p27 Kip1 (Figure 5). Importantly, UVB modified the molecular response on exposure to KYN or KYNA in melanoma SK-MEL-3 cells. Interestingly, UVB alone stimulated the protein level of cyclin D1 and modified the biological response to tryptophan metabolites in SK-MEL-3 cells (Figure 5). UVB induced the protein expression of p21 Waf1/Cip1, but the stimulatory effect of KYN and KYNA was no longer observed in SK-MEL-3 cells exposed previously to UVB (Figure 5). Interestingly, we observed the opposite effect of KYNA (5 mM) on p27 Kip1 in SK-MEL-3 cells exposed and not exposed to UVB. As it was mentioned above, KYNA (5 mM) stimulated p27 Kip1 under standard conditions, whereas it decreased the protein level of this inhibitor of cyclin-dependent kinase in SK-MEL-3 cells exposed previously to UVB (Figure 5b). We also studied the effect of KYN and KYNA on the activation of ERK 1/2 signaling kinase. Both tryptophan metabolites did not affect the phosphorylation of ERK1/2 under standard conditions, whereas KYN and KYNA stimulated ERK1/2 activation in SK-MEL-3 cells previously exposed to UVB (Figure 5).

**Figure 6:**
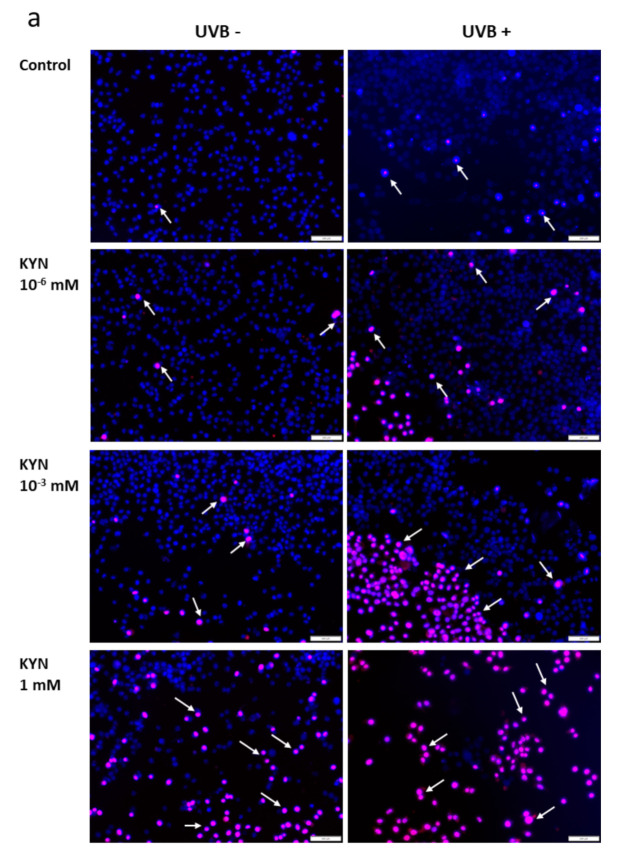

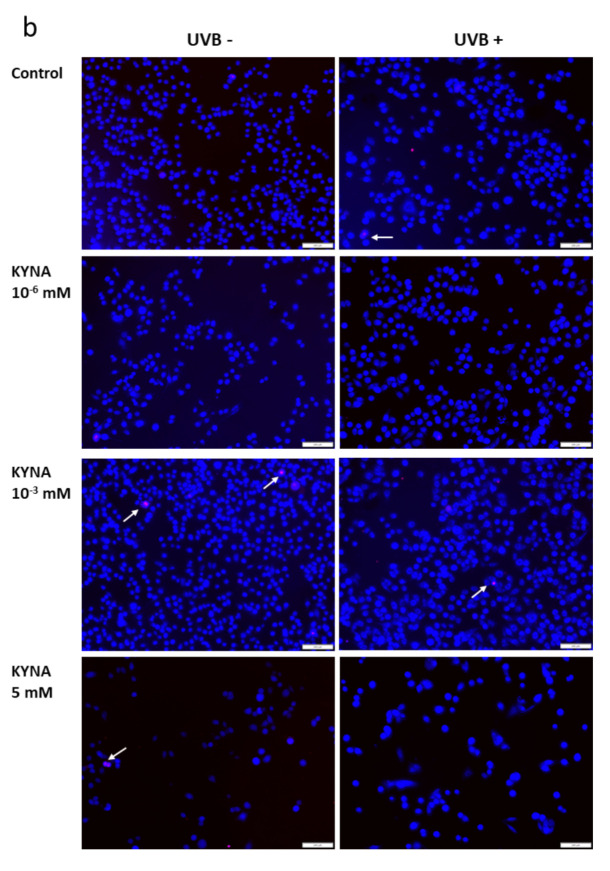

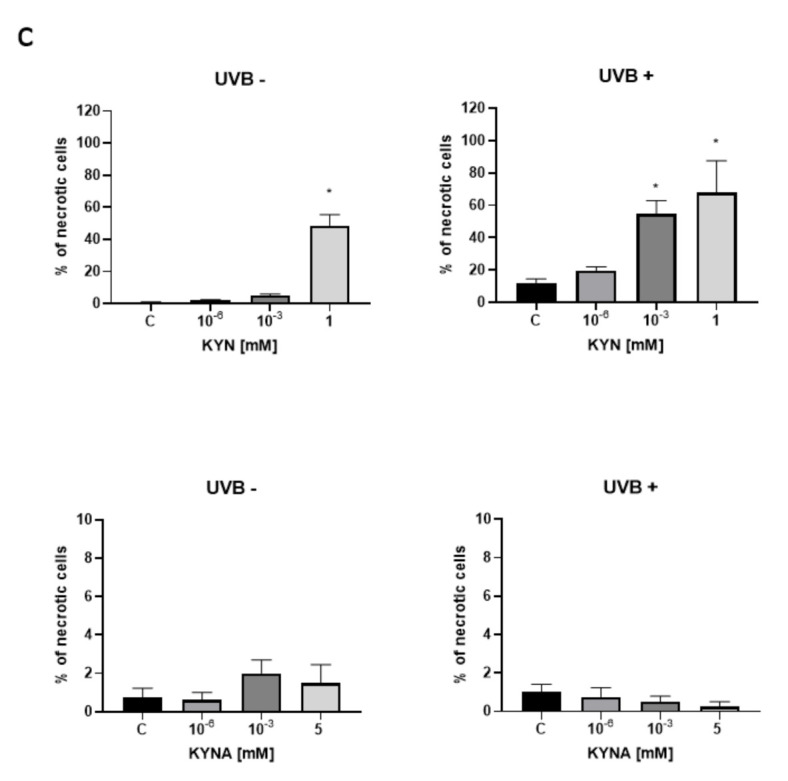
The effect of UVB on cell death in melanoma SK-MEL-3 cells exposed to KYN (**a**) and KYNA (**b**). Melanoma SK-MEL-3 cells were exposed to UVB (20 mJ/cm^2^; UVB+) and then to culture medium (control, C) or serial dilutions of KYN (10^−6^, 10^−3^, and 1 mM) and KYNA (10^−6^, 10^−3^, and 5 mM) for 24 h. Similarly, the biological activity of KYN and KYNA was studied in melanoma cells under standard conditions (UVB−). The effect of UVB and tryptophan metabolites on the induction of cell death was assessed by co-staining with Hoechst 33342 (blue) and propidium iodide (red). Apoptotic fraction represented cells with fragmented nuclei stained in intense blue color. Nuclei of necrotic cells were stained with pink color (indicated by white arrows). Magnification 10×. (**c**) Quantitative analysis of necrotic cells. The number of necrotic cells per 100 SK-MEL-3 cells was assessed in at least three fields of view. Values significant (*) in comparison with the control with *p* < 0.05 (one-way ANOVA with Tukey’s post hoc test).

The effect of KYN and KYNA on the induction of cell death was assessed by co-staining with Hoechst 33342 and propidium iodide. A similar experiment was also performed after exposure of melanoma SK-MEL-3 cells to UVB. Unfortunately, KYN at a concentration of 5 mM significantly decreased the proliferation and adhesion of SK-MEL-3 cells; thus the highest concentration of this tryptophan metabolite used in this experiment was 1 mM. Importantly, fluorescent staining indicated that KYN at a concentration of 1 mM induced necrosis in SK-MEL-3 cells (Figure 6a,c). Moreover, this effect was enhanced in melanoma cells exposed to UVB. A significant increase in necrotic cell fraction was observed in UVB−treated SK-MEL-3 cells exposed to 10^−3^ and 1 mM KYN (Figure 6c). On the other hand, despite the evident reduction of cell number, even the highest tested concentration of KYNA did not affect cell death in SK-MEL-3 cells under standard conditions and after UVB exposure (Figure 6b,c). No significant changes in the apoptotic cell fraction were observed in SK-MEL-3 cells incubated with KYN or KYNA under standard conditions and after UVB exposure.

## 3. Discussion

In this study, for the first time, we showed the effect of UVB on the biological activity of tryptophan metabolites KYN and KYNA toward melanoma cells in vitro. We revealed the effect of UVB on the proliferation and invasiveness of melanoma A375, SK-MEL-3, and RPMI-7951 cells. Taking into consideration that the skin is constantly exposed to endogenous and exogenous tryptophan metabolites, AhR ligands KYN and KYNA, and to genotoxic and mutagenic UVB radiation [23], these results may have a huge impact on society. KYN and KYNA are endogenously synthesized in the human body. Their presence in various body fluids and tissues was previously reported (reviewed in [4,15,16]). Previous studies confirmed the presence of KYN and KYNA in skin cells [24]. The basal level of KYN was estimated at 0.24 ± 0.013 μM in skin fibroblasts and 0.51 ± 0.027 μM in keratinocytes, whereas KYNA was present at a concentration of 3.70 ± 0.20 μM in skin fibroblasts and 2.7 ± 0.13 μM in keratinocytes [24]. Moreover, selected tryptophan metabolites were also found in human sweat [25]. Bearing in mind the exogenous sources of KYN and KYNA in the human body, it should be noted that these tryptophan metabolites might be absorbed from the gastrointestinal tract and distributed to the periphery as they are found in various beverages and food products [18,19,21,22,26,27,28,29,30]. Interestingly, previous studies revealed that KYNA is also present in plants, including herbs, and in bee products used in cosmetics and skin care treatments. Unfortunately, there are limited data concerning the level of selected tryptophan metabolites that may reach the skin cells after oral administration. On the other hand, taking into consideration the evaporation and condensation of sweat, as well as the frequent use of several cosmetics and herbs containing tested compounds in daily care treatments, skin cells may be exposed to much higher doses of tryptophan metabolites than found in skin cells. Thus, in this study, we focused on the biological activity of a wide range of concentrations of KYN and KYNA, representing physiological concentrations as well as high millimolar concentrations. Moreover, previous studies revealed that only KYN at a concentration at 5 mM significantly inhibited the proliferation of normal melanocytes in vitro [11].

The effect of tryptophan-derived AhR ligands KYN and KYNA on melanomagenesis and melanoma progression has not been fully elucidated. Despite the fact that AhR plays an important role in various physiological processes in the skin [5,31], previous studies indicated the possible involvement of this receptor in skin cancer formation and progression. Long-term observations revealed that overexposure to some synthetic AhR ligands (i.e., polycyclic aromatic hydrocarbons) or UVB might lead to premalignant lesions or skin cancer [5,32,33]. Although some of the AhR ligands, including 2,3,7,8-tetrachlorodibenzo-*p*-dioxin (TCDD), exert procarcinogenic properties [34,35], and the biological effects of the activation of some AhR-target genes may suggest the involvement of this receptor in tumor progression [36], a specific role of AhR in carcinogenesis has not been fully elucidated. Previous studies revealed that the involvement of AhR in cell migration is cell-type-dependent and might be responsible for some contradictory functions of this receptor in carcinogenesis [37]. Interestingly, AhR may play the role of tumor suppressor in melanoma. Contador-Troca et al. reported that AhR expressed in melanoma cells was involved in the inhibition of tumor growth and metastasis. However, its expression in the stroma promoted melanomagenesis [37]. Recently, we reported that selected tryptophan metabolites affected the DNA synthesis, cytotoxicity and cell death of melanoma A375 and RPMI-7951 cells, representing subsequent stages of cancer progression [11]. Similarly, in the present study, we revealed the significant inhibitory activity of KYN on DNA synthesis in SK-MEL-3 cells (Figure 1c). However, KYNA did not affect DNA synthesis in either A375 or RPMI-7951 cells [11]. Interestingly, KYNA at the highest tested concentration of 5 mM moderately decreased DNA synthesis in SK-MEL-3 cells (Figure 1d). The genetic diversity of the melanoma cell lines should be emphasized. SK-MEL-3 and RPMI-7951 cells represent metastatic melanoma, in contrast to A375 cells derived from primary melanoma [38]. SK-MEL-3 cells bear the mutation in *BRAF* (p.V600E) and *PT53* genes (cancer cell line mutation data [38]). However, unlike A375 and RPMI cells, SK-MEL-3 cells have the functional form of the *CDKN2A* gene (cancer cell line mutation data [38]), which may be crucial for KYNA biological activity toward melanoma cells.

Importantly, we revealed that UVB itself decreased DNA synthesis and acted additively to tryptophan metabolites in inhibiting the proliferation of melanoma cells in vitro; however, the effect was dependent on the type of melanoma cell line and the concentration of tested substances (Figure 1). Moreover, the antiproliferative activity of KYNA was observed only in A375 and RPMI-7951 cells exposed to UVB (Figure 1b,f), which may suggest that UVB sensitized the melanoma A375 and RPMI-7951 cells to KYNA activity. The possible interactions between UVB and tryptophan metabolites KYN and KYNA have not been fully elucidated. Previous studies reported the upregulation of IDO, the key enzyme in KYN synthesis, by some proinflammatory cytokines (including IFN-γ or TNF-α), which in turn may be triggered in the skin upon UVB exposure [39]. Interestingly, Sheipouri et al. showed that UVB induced the production of KYN and KYNA in skin fibroblasts and keratinocytes. Moreover, UVB decreased the intracellular NAD^+^ level and the viability of these skin cells [24]. Thus, it cannot be excluded that UVB might also induce significant changes in the metabolism of melanoma cells, which might modify the biological response of UVB−treated melanoma cells to KYN and KYNA. Moreover, KYNA was considered a phototoxic agent toward erythrocytes and glial cells; however, no specific studies have been conducted on skin cells [40,41].

In this study, we showed that UVB and tested tryptophan metabolites did not affect the invasiveness of melanoma A375 and RPMI-7951 cells, representing different stages of melanomagenesis (Figure 2a,c). Surprisingly, SK-MEL-3 cells had a different biological response profile (Figure 2b). Under standard conditions, only KYN stimulated the migration and invasiveness of melanoma SK-MEL-3 cells. However, after UVB exposure, both tryptophan metabolites increased the invasiveness of SK-MEL-3 cells. Interestingly, UVB itself moderately decreased the migration and invasiveness of melanoma SK-MEL-3 cells (Figure 2b). However, the molecular mechanism of this activity in SK-MEL-3 cells has not been fully revealed. Both tryptophan metabolites, KYN and KYNA, did not significantly affect the protein level of E-cadherin, N-cadherin, and β-catenin, which are involved in the migration and adhesion (Figure 3a,b). Importantly, previous studies suggested that β-catenin may be considered an essential survival factor for melanoma metastasis [42]. On the other hand, it cannot be excluded that this characteristic response of SK-MEL-3 cells to tryptophan metabolites (Figure 2b) resulted from specific UVB−induced molecular alterations in these cells. Functional *CDKN2A* and *PTEN* genes are characteristic features of SK-MEL-3 cells among all tested melanoma cell lines. Importantly, previous studies revealed the potential interactions between *CDKN2A* and *PTEN* genes and UVB radiation. *CDKN2A* may be involved in the proliferation and migration of melanoma cells, as the inhibitory effect was observed in *CDKN2A*-overexpressed melanoma A375 cells [43]. However, Krähn et al. reported the inhibitory effect of UVB on *CDKN2A* expression in melanoma samples [44]. Similarly, previous studies revealed the potential interaction between PTEN and UVB radiation mediated by signaling kinases [45,46]. However, we did not observe any significant changes in the activation of ERK1/2 kinase in SK-MEL-3 cells in response to UVB exposure (C UVB− vs. C UVB+; Figure 5). Although the potential involvement of the *CDKN2A* and *PTEN* genes cannot be excluded, it does not directly explain the observed effect of UVB and tryptophan metabolites on the migration and invasiveness of melanoma SK-MEL-3 cells. Therefore, it remains unresolved whether, indeed, tryptophan metabolites induce the migration of SK-MEL-3 cells after UVB exposure or KYN and KYNA create such an unfavorable environment for cancer cells that induces their migration through the endothelium in vitro.

Due to the different biological activities of KYN and KYNA toward melanoma SK-MEL-3 cells, we decided to study the effect of tryptophan metabolites on metabolic activity, cell cycle regulation, and cell death in this melanoma cell line. The majority of the results confirmed the anticancer activity of KYN and KYNA toward melanoma SK-MEL-3 cells. Both tryptophan metabolites inhibited the metabolic activity of SK-MEL-3 cells (Figure 4). Moreover, UVB significantly enhanced the negative effect of KYN and KYNA (Figure 4). In this study, we confirmed that KYNA affected more effectively the metabolic activity of melanoma cells rather than DNA synthesis (Figure 1d). A similar effect was observed in previously published studies on glioma cells, colon cancer, and renal cancer cells [10,13,14]. Although KYN and KYNA belong to the same group of tryptophan metabolites, differences in the effectiveness of inhibition of metabolic activity of SK-MEL-3 cells were observed (Figure 4). KYNA at a concentration of 1 mM and 5 mM decreased metabolic activity by 32% and 58%, respectively, whereas the inhibitory potential of KYN was observed only at a concentration of 5 mM (Figure 4).

The antiproliferative potential of tryptophan metabolites KYN and KYNA toward SK-MEL-3 cells might have resulted from their effect on inhibitors of cyclin-dependent kinases (CDK). Both tryptophan metabolites increased the protein level of p21 Waf1/Cip1 and p27 Kip1 (Figure 5). Importantly, previous studies revealed that the expression of p21 Waf1/Cip1 was significantly decreased in approximately 30% of primary melanomas and in 40% of metastases. Moreover, it was suggested that alteration in p21 Waf1/Cip1 expression might be crucial for melanoma promotion and progression [47]. Thus, the stimulatory activity of KYN and KYNA on the protein level of these CDK inhibitors may play a significant role in the antiproliferative activity of tested tryptophan metabolites toward melanoma SK-MEL-3 cells under standard conditions. A similar effect of KYNA on the p21 Waf1/Cip1 protein level was previously described in colon cancer HT-29 cells [13]. Importantly, UVB increased the protein level of p21 Waf1/Cip1 and p27 Kip1 in melanoma SK-MEL-3 (Figure 5). This may be the reason for the decreased proliferation of SK-MEL-3 cells after UVB exposure (Figure 1c,d). Interestingly, the protein level of p27 Kip1 was decreased in comparison with the control (UVB+) in SK-MEL-3 cells exposed to UVB and KYNA (5 mM) (Figure 4b), but this did not stimulate proliferation (Figure 1d). It is not excluded that other molecular mechanisms might be involved in the antiproliferative activity of tryptophan metabolites under UVB conditions.

Moreover, we revealed that KYN stimulated necrosis in SK-MEL-3 cells and this process was enhanced by UVB (Figure 6a). Interestingly, this effect was not observed after exposure to KYNA (Figure 6b). Recent studies reported that tryptophan metabolites, including KYN and KYNA, induced cell death in melanoma A375, but not in RPMI-7951 cells [11]. Despite the same origin of melanoma SK-MEL-3 and RPMI7951 cells (lymph node metastasis) [38,48], we reported increased necrosis in SK-MEL-3 cells, whereas this tryptophan metabolite did not affect cell death in RPMI-7951 cells [11]. The reason for this phenomenon may be genetic differences between these two melanoma cell lines. Unlike RPMI-7951 cells, SK-MEL-3 cells have the functional form of the *CDKN2A* and *PTEN* genes.

On the other hand, some of the results regarding the influence of tryptophan metabolites on the regulation of the cell cycle and signaling pathways in SK-MEL-3 cells are disturbing. KYN and KYNA at a concentration of 5 mM increased the level of cyclin D1 in SK-MEL-3 cells (Figure 5). However, differences in the biological activities of these compounds in SK-MEL-3 cells were observed after exposure to UVB. UVB itself increased the protein level of cyclin D1 (Figure 5). These molecular alterations might have resulted in modified biological responses of melanoma SK-MEL-3 cells to KYN and KYNA under standard conditions and after exposure to UVB.

Moreover, we revealed that KYN and KYNA activated ERK1/2 kinase in melanoma SK-MEL-3 cells after UVB exposure, but not under standard conditions (Figure 5). ERK1/2 is the main kinase in the Ras/Raf/MEK/ERK signaling pathway involved in the various cellular processes including not only proliferation, differentiation, adhesion, and cellular senescence, but also migration and adhesion [49]. In the present study, activation of ERK1/2 might have led to cyclin D1 upregulation in SK-MEL-3 cells exposed to UVB (Figure 5). Moreover, Villanueva et al. suggested that ERK1/2 activity may also be involved in p27 Kip1 downregulation mediated by Skp2 [50]. However, taking into consideration the stimulatory potential of UVB on the antiproliferative activity of KYN and KYNA toward SK-MEL-3 cells, the activation of ERK1/2 and upregulation of cyclin D1 in SK-MEL-3 cells exposed to UVB resulted from a strong stress stimulus. On the other hand, it cannot be excluded that the activation of ERK1/2 by tryptophan metabolites was involved in enhanced invasiveness of SK-MEL-3 cells after exposure to UVB (Figure 2b).

It should be noted that KYN is a precursor of other metabolites of the kynurenine pathway, including KYNA. However, the observed biological effects of both tested compounds on melanoma cells were different. These results may suggest that KYN has not been completely converted into KYNA in these experiments. On the other hand, it cannot be excluded that exogenous KYN and KYNA might interact with the endogenous kynurenine pathway in melanoma cells.

This study focused on the potential biological interactions between selected tryptophan metabolites and UVB radiation in melanoma cells. However, it should be noted that tryptophan metabolites may also be involved in the activation of the immune system. KYN is considered to be the most potent immunosuppressant among tryptophan metabolites and it may play a crucial role in cancer immune escape and tumor resistance to immunotherapy [51]. Additionally, Labadie et al. reported that the tryptophan–KYN–AhR pathway may be involved in the immunosuppression of T cell-inflamed tumors, including melanoma [52]. On the other hand, several studies confirmed the antiproliferative properties of KYNA toward various cancer cell types [4,9,10,11,12,13,14]. However, Rad Pour et al. suggested that lowered activity of kynurenine 3-monooxygenase and higher KYNA production might be alternative immune regulatory mechanisms in melanoma, leading to dysfunctional effector CD4+ T cell response [53]. Therefore, further advanced studies concerning the potential effect of UVB on the immune activity of tryptophan metabolites would be valuable.

To conclude, in the present study, we showed for the first time the effect of UVB on the biological activity of selected tryptophan metabolites, KYN and KYNA, toward melanoma cells in vitro. These results may have a significant impact, considering the frequent exposure of the human skin to UVB and to endogenous and exogenous sources of tryptophan metabolites. We showed that UVB enhances the antiproliferative activity of both tryptophan metabolites KYN and KYNA in melanoma A375, SK-MEL-3 and RPMI-7951 cells, which may be related to the increased protein level of p21 Waf1/Cip1 and p27 Kip1 in melanoma cells exposed to UVB. Additionally, UVB increases the inhibitory activity of KYN and KYNA on the metabolic activity of melanoma SK-MEL-3 cells in vitro and it enhances KYN-induced necrosis in SK-MEL-3 cells. On the other hand, certain molecular and biological responses to UVB, including increased invasiveness or cyclin D1 and ERK1/2 upregulation in SK-MEL-3 cells, are disturbing and need further studies. The results suggest that UVB may sensitize melanoma cells to the effects of selected tryptophan metabolites; however, various molecular mechanisms may play a role in this process. UVB is also considered a proinflammatory factor and may affect the kynurenine pathway and NAD (+) production in skin fibroblasts and keratinocytes [24]. It cannot be excluded that similar changes may also be observed in melanoma cells exposed to UVB. On the other hand, the biological activity of KYN and KYNA may not be strictly dependent on the AhR receptor. It should be underlined that despite belonging to the same group of AhR ligands, both tryptophan metabolites KYN and KYNA may exert non-receptor activity. Moreover, KYNA is considered a G-protein-coupled receptor 35 (GPR35) agonist [54] and an antagonist of glutamate receptors and α7 nicotinic acetylcholine receptor [55,56,57].

In our studies, we did not observe significant biological activity of selected tryptophan metabolites toward melanoma cells in vitro at low physiological concentrations. Interestingly, high millimolar concentrations of KYN and KYNA exerted anticancer activity, but the effect was dependent on cell type. Therefore, further advanced in vivo and clinical studies are necessary to determine whether the selected tryptophan metabolites may play a role in supporting the standard anticancer therapy of melanoma.

## 4. Materials and Methods

### 4.1. Drugs

L-KYN (L-isomer of KYN), obtained from Sigma-Aldrich (St. Louis, MO, USA), was dissolved in cell culture medium. KYNA, purchased from Sigma-Aldrich (St. Louis, MO, USA), was dissolved in 1 N NaOH and phosphate buffered saline (PBS; Sigma-Aldrich, St. Louis, MO, USA). No significant effects of solvents on proliferation and morphology were observed in melanoma A375, SK-MEL-3, and RPMI-7951 cells.

### 4.2. Cell Cultures

Human melanoma A375, SK-MEL-3, and RPMI-7951 cells were obtained from the American Type Culture Collection (ATCC; Manassas, VA, USA). A375 cells were maintained in Dulbecco’s modified Eagle’s medium (DMEM) supplemented with 10% heat-inactivated fetal bovine serum (FBS). SK-MEL-3 cells were cultured in McCoy’s 5A modified medium supplemented with 15% FBS, whereas RPMI 7951 cells were maintained in minimum essential medium with Earle′s salts supplemented with sodium pyruvate (final concentration 1 mM) and 10% FBS. The culture medium was supplemented with penicillin (100 U/mL) and streptomycin (100 µg/mL). Human umbilical vein endothelial cells (HUVEC), obtained from Sigma-Aldrich (St. Louis, MO, USA), were grown in endothelial cell growth medium. Cells were maintained in a humidified atmosphere composed of 95% air and 5% CO_2_ at 37 °C. All cell culture reagents were purchased from Sigma-Aldrich (St. Louis, MO, USA).

### 4.3. Experiment Design

Human melanoma A375, SK-MEL-3, and RPMI-7951 cells were exposed to Hanks’ balanced salt solution (HBSS; Sigma-Aldrich, St. Louis, MO, USA) (control, C) or serial dilutions of KYN and KYNA dissolved in HBSS for 1 h in a humidified atmosphere of 95% air and 5% CO_2_ at 37 °C. Then, HBSS was discarded and cells were exposed to a fresh culture medium (control, C) or serial dilutions of KYN or KYNA in a fresh culture medium and incubated for 23 h in a humidified atmosphere of 95% air and 5% CO_2_ at 37 °C. This experiment design provided the maximal bioavailability of KYN and KYNA and reduced the possible interactions between tested tryptophan metabolites and tryptophan included in the cell culture medium. The above description relates to the standard conditions of the experiment (without UVB exposure; UVB−). To study the effect of UVB radiation on the biological activity of KYN and KYNA, melanoma A375, SK-MEL-3 and RPMI-7951 cells were exposed to UVB (A375: 11 mJ/cm^2^; SK-MEL-3 and RPMI-7951: 20 mJ/cm^2^). Then, HBSS (control) or serial dilutions of KYN and KYNA in HBSS were added. Melanoma cells were incubated for 1 h in a humidified atmosphere of 95% air and 5% CO_2_ at 37 °C. Next, HBSS was discarded and cells were exposed to a fresh culture medium (control, C) or serial dilutions of KYN or KYNA in a fresh culture medium and incubated for 23 h in a humidified atmosphere of 95% air and 5% CO_2_ at 37 °C. Then, further tests were performed (BrdU Assay, MTT Assay, Western blot, Tumor Cell Transendothelial Migration Assay, Hoechst 33324, and PI staining). UVB exposure was performed using an automated irradiation system, BIO-SUN (wavelength: 312 nm; time of the exposure < 1 min; irradiation distance: 25 mm; Vilber Lourmat Deutschland GmbH, Eberhardzell, Germany). The dose of UVB was determined in the preliminary experiments. Briefly, melanoma cells were exposed to various doses of UVB (11, 20, 50, 100, and 500 mJ/cm^2^). Morphological changes were observed after 1, 2, 4, 8, and 24 h incubation. Additionally, the effect of different doses of UVB was determined by the MTT Assay after 24 h incubation.

### 4.4. BrdU Assay

The BrdU Assay was performed to determine the effect of KYN and KYNA on the proliferation of melanoma A375, SK-MEL-3, and RPMI-7951 cells according to a previously described procedure [10]. The BrdU Assay is based on the measurement of 5′-bromo-2′-deoxy-uridine (BrdU) incorporation into the newly synthesized DNA of actively proliferating cells. Briefly, melanoma cells were plated in 96-well plates (NUNC, Roskilde, Denmark) at a density of 2 × 10^4^ cells/mL (A375) or 4 × 10^4^ cells/mL (SK-MEL-3, RPMI-7951). Next day, the cells were treated with serial dilutions of tested compounds (KYN: 10^−9^, 10^−6^, 10^−3^, 1, and 5 mM; KYNA: 10^−9^, 10^−6^, 10^−3^, 1, and 5 mM) or a fresh cell culture medium (control, C) according to the experiment design described in detail above (standard conditions). A similar experiment was performed on melanoma cells previously exposed to UVB. Cell proliferation was assessed after 24 h incubation according to the manufacturer’s protocol (Cell Proliferation ELISA BrdU, Roche Diagnostics GmbH, Penzberg, Germany).

### 4.5. Tumor Cell Transendothelial Migration Assay

Tumor Cell Transendothelial Migration Assay (Millipore’s QCM Tumor Cell Transendothelial Migration Assay—Colorimetric; Merck Millipore, Burlington, MA, USA) was applied to study the effect of KYN, KYNA, and UVB on the ability of melanoma A375, SK-MEL-3, and RPMI-7951 cells to invade the endothelium. The suspension of HUVEC endothelial cells (1 × 10^5^ cells) was added to each insert. The cells were grown for 48 h in a humidified atmosphere of 95% air and 5% CO_2_ at 37 °C (to reach ~95% confluence). Then, the inserts were transferred to new wells containing 300 μL of serum-free melanoma culture medium supplemented with 0.5% bovine serum albumin (BSA). A375, SK-MEL-3, and RPMI-7951 cells were exposed to UVB, KYN (1 mM), and KYNA (5 mM) for 24 h according to the procedure described in detail above. Unfortunately, KYN at a concentration of 5 mM induced significant morphological changes in HUVEC cells and disrupted the endothelial monolayer. Thus, the highest concentration of KYN used in this experiment was 1 mM. Then, melanoma cells (1 × 10^5^ cells) in serum-free melanoma culture medium supplemented with 0.5% BSA (control, C) or the dilution of KYN (1 mM) or KYNA (5 mM) were added to the upper well of the inserts. The negative controls for this assay comprised inserts with HUVECs cell alone (no tumor cells control) and cell culture medium only (no cells control). Inserts were incubated in regular conditions for 24 h, and after that, melanoma cells from the underside of the inserts were stained, extracted, and colorimetrically quantified according to the manufacturer’s instructions. The absorbance was proportional to the migration of tumor cells.

### 4.6. MTT Assay

The MTT Assay assesses cellular metabolic activity. The MTT Assay is based on the reduction of a tetrazolium salt (3-(4,5-dimethylthiazol-2-yl)-2,5-diphenyltetrazolium bromide, MTT) into an insoluble formazan product by metabolically active cells. SK-MEL-3 cells were plated in 96-well plates (Nunc, Roskilde, Denmark) at a density of 4 × 10^4^ cells/mL. Next day, the cells were exposed to serial dilutions of tested compound (KYN: 1, and 5 mM; KYNA: 1, and 5 mM) or a fresh cell culture medium (control, C) for 96 h according to the experiment design described in detail above (standard conditions). A similar experiment was performed in SK-MEL-3 cells previously exposed to UVB (20 mJ/cm^2^). Then, the cells were incubated for 3 h with MTT solution (5 mg/mL) in a humidified atmosphere of 95% air and 5% CO_2_ at 37 °C. After the incubation time, the cells were incubated with sodium dodecyl sulfate (SDS) buffer (10% SDS in 0.01 N HCl) overnight. The absorbance was measured at 570 nm (microplate reader (Epoch, BioTek Instruments, Inc., Winooski, VT, USA) with Gen5 software (v. 2.01, BioTek Instruments, Inc., Winooski, VT, USA).

### 4.7. Western Blot

Melanoma SK-MEL-3 cells were exposed to serial dilutions of tested compounds (10^−9^, 10^−6^, 10^−3^, 1, and 5 mM) or a fresh cell culture medium (control, C) for 24 h according to the experimental design described in detail above. The protein expression or its activation was assessed by Western blot according to the procedure previously described in [11]. The following primary antibodies were used in the procedure: cyclin D1, CDK4, p21 Waf1/Cip1, p27 Kip1, E-cadherin, N-cadherin, β-catenin, phospho-ERK1/2, and β-actin antibody (1:1000; Cell Signaling Technology, Danvers, MA, USA). The following secondary antibodies coupled to horseradish peroxidase were used: anti-rabbit IgG, HRP-linked Antibody and anti-mouse IgG, HRP-linked Antibody (1:2000) (Cell Signaling Technology, Danvers, MA, USA). The visualization of the bands was performed using an enhanced chemiluminescence detection system (Pierce Biotechnology, Waltham, MA, USA) and the Syngene G:BOX Chemi XT4 gel documentation system (Syngene, Cambridge, UK).

### 4.8. Fluorescent Cell Death Analysis

Briefly, melanoma SK-MEL-3 cells were seeded on Lab-Tek Chamber Slide (Nunc, Roskilde, Denmark) at a density of 6 × 10^4^ cells/mL the day before the treatments as per the experimental design described above (KYN: 10^−6^, and 10^−3^, 1 mM; KYNA: 10^−6^, 10^−3^, and 5 mM; UVB: 20 mJ/cm^2^). After 24 h incubation, the effect of KYN and KYNA on induction of cell death was analyzed after fluorescence staining with Hoechst 33342 and propidium iodide as mentioned in detail previously [11].

Visualization was performed using fluorescence microscopy (Olympus IX83 System Microscope; Olympus Optical Co. Ltd. and CellSens RT software, Olympus Optical Co., Ltd., Tokyo, Japan) at 10× magnification.

### 4.9. Data Analysis

The data were shown as the mean value ± standard error of the mean (SEM) and statistically analyzed using one-way ANOVA with Tukey’s post hoc test or unpaired *t*-test (significance was accepted at *p* < 0.05) (GraphPad Prism 8 software; GraphPad Software, Inc., La Jolla, CA, USA). Densitometric analysis of Western blots was performed using NIH ImageJ software (Wayne Rasband, Bethesda, MD, USA). Numerical data, normalized relative to β-actin, are shown as relative value of the control (the fold changes in protein expression ≥ 30% were considered significant; qualitative analysis).

## Data Availability

The data presented in this study are available in insert article.

## Abbreviations

AhR	aryl hydrocarbon receptor
BrdU	5′-bromo-2′-deoxy-uridine
BSA	bovine serum albumin
CDK	cyclin-dependent kinase
CPD	cyclobutane pyrimidine dimers
FBS	fetal bovine serum
GPR35	G-protein-coupled receptor 35
HBSS	Hanks’ balanced salt solution
HUVEC	human umbilical vein endothelial cells
KYN	kynurenine
KYNA	kynurenic acid
MMP	matrix metalloproteinase
MTT	3-(4,5-dimethylthiazol-2-yl)-2,5-diphenyltetrazolium bromide
PBS	phosphate buffered saline
ROS	reactive oxygen species
SDS	sodium dodecyl sulfate
SEM	standard error of the mean
TCDD	2,3,7,8-tetrachlorodibenzo-*p*-dioxin
